# Heroin Overdose Complicated by Compartment Syndrome, Rhabdomyolysis, and Acute Renal Failure

**DOI:** 10.7759/cureus.61144

**Published:** 2024-05-26

**Authors:** Kevin Tom, Jeffrey Bzdusek

**Affiliations:** 1 Emergency Medicine, Endeavor Health Swedish Hospital, Chicago, USA; 2 Emergency Medicine, St. Bernard Hospital, Chicago, USA

**Keywords:** acute rhabdomyolysis, acute renal failure, lower extremity compartment syndrome, heroin overdose, opioid-abuse

## Abstract

The opioid-abuse epidemic is a problem that continues to persist world-wide. As such, appropriately evaluating and treating such patients is crucial, especially when considering the various complications that may arise. In rare cases, opioid overdoses can be complicated by compartment syndrome, rhabdomyolysis, and acute renal failure. All three of these complications can result in life threatening emergencies.

We present a case of a 38-year-old male who was brought to the emergency department after reportedly being found lying on the ground for an unknown period of time from suspected heroin overdose. He was initially treated with 2 milligrams (mg) of intramuscular naloxone en route via emergency medical services with appropriate response. Shortly after arrival to the emergency department, the patient complained of severe right lower extremity pain, paresthesia and paralysis. Patient developed acute lower extremity compartment syndrome that was further complicated by rhabdomyolysis and acute renal failure.

While emergency medicine physicians are familiar with the common complications of heroin overdose including mental status changes, respiratory depression and gastrointestinal symptoms, they must also be familiar with the less common ones. Notably, acute compartment syndrome. Compartment syndrome is ultimately a clinical diagnosis and warrants emergent surgical consultation. Every patient presenting to the emergency department warrants a complete, thorough physical examination to evaluate for any and all life-threatening conditions, regardless of the presenting complaint.

## Introduction

Over the last two decades, the opioid epidemic has only continued to worsen. With opioid-related deaths having quadrupled since the early 2000s, it is important to appropriately evaluate and treat such patients by considering all potential complications [1]. Patients presenting to the emergency department after an opioid overdose may demonstrate neurologic, respiratory, and gastrointestinal symptoms. It is important, however, to also consider orthopedic emergencies including compartment syndrome.

Compartment syndrome is a life-threatening orthopedic emergency that occurs when increasing pressure in an enclosed myofascial compartment results in impaired local circulation leading to muscle ischemia and necrosis [2]. It is generally considered a clinical diagnosis; however, it can also be definitively diagnosed with an intra-compartmental pressure >30 millimeters of mercury (mmHg) [2-4]. The majority of acute compartment syndrome cases are due to fractures (75%); other common causes include penetrating/blunt trauma, burns, vascular injury, and infection [3-5]. In rare circumstances, opioid overdoses can be complicated by acute compartment syndrome, rhabdomyolysis, and even acute renal failure [2,5,6]. We present a case report of this below.

## Case presentation

A 38-year-old male was brought to the emergency department after a suspected heroin overdose. According to emergency medical services (EMS), the patient was found lying on the ground in an apartment complex courtyard for an unknown period of time and was minimally responsive with pinpoint pupils. The patient was administered 2 milligrams (mg) of intramuscular (IM) naloxone and immediately became awake, alert, and responsive to questioning. The patient reported snorting heroin earlier that day but is unsure exactly how many hours prior.

The patient reported no significant medical history, allergies, or surgical history. He denied taking any prescription or over-the-counter medications. Personal social history was significant for daily heroin use. 

On arrival at the emergency department, the patient’s vital signs were as follows: maximum temperature 96.7 degrees Fahrenheit (°F), respiratory rate 14 breaths per minute, heart rate 57 beats per minute (bpm), blood pressure 100/52 mmHg, 98% oxygen saturation on room air. The patient arrived alert, oriented (to person, place, and time), and was answering questions appropriately. He denied any complaints on arrival and requested to sleep. Pupils were equal, round, and reactive to light. The patient appeared well, in no distress, and was not clinically intoxicated. The treatment plan was to monitor this patient clinically for two to four hours for common signs of recurrent opioid intoxication, mental status changes, respiratory distress, and signs of withdrawal.

The patient was re-evaluated after a seemingly uncomplicated course in the emergency department three hours later at which point the patient was awakened from sleep. At this point, the patient began to complain of right lower extremity paresthesia, paralysis, and pain. On re-examination, the patient was noted to have an extremely firm, tender, and circumferentially swollen right lower extremity distal to the right knee. The patient did not have an obvious palpable dorsalis pedis or posterior tibial pulse.

Initial laboratory tests were drawn and are illustrated below in Table 1. Notable lab results include white blood cell (WBC) = 19.4 x 103/uL, potassium (K) = 8.0 meq/L, creatine kinase (CK) = 212,845 U/L, blood urea nitrogen (BUN) = 34 mg/dL, creatinine (Cr) = 3.0 mg/dL, aspartate transaminase (AST) = 1467 U/L, alanine transaminase (ALT) = 537 U/L, and lactic acid (LA) = 4.9 mmol/L. Urinalysis (UA) demonstrated tea-colored urine with large amounts of blood and no red blood cells (RBC).

**Table 1:** Laboratory values

Laboratory test (units)	Patient value	Reference range
White blood cell (10^3^/uL)	19.4	4.50 – 13.0
Red blood cell (10^6^/ uL)	5.74	4.20 – 6.00
Hemoglobin (g/dL)	16.7	13.5 – 18.0
Hematocrit (%)	50.8	40.0 – 54.0
Platelets (10^3^/uL)	265	130 – 500
Glucose (mg/dL)	252	70 – 105
Sodium (meq/L)	137	135 – 147
Potassium (meq/L)	8.0	3.4 – 5.3
Chloride (meq/L)	101	96 – 108
Carbon dioxide (meq/L)	26	22 – 32
Glomerular filtration rate (mL/min)	18	60 – 999
Blood urea nitrogen (mg/dL)	34	7 – 23
Creatinine (mg/dL)	3.0	0.4 – 1.3
Calcium (mg/dL)	6.6	8.6 – 10.5
Magnesium (mg/dL)	1.8	1.6 – 2.6
Phosphorus (mg/dL)	4.8	2.6 – 5.0
Albumin (g/dL)	4.0	3.5 – 5.0
Total bilirubin (mg/dL)	0.5	0.2 – 1.4
Alkaline Phosphatase (U/L)	91	46 – 116
Aspartate transaminase (U/L)	1467	5 – 34
Alanine transaminase (U/L)	537	7 – 50
International normalized ratio	1.07	0.8 – 1.2
Prothrombin time (s)	13.8	11 – 13.5
Partial thromboplastin time (s)	24.3	25 – 35
Creatine kinase (U/L)	212,845	46 – 171
Lactic acid (mmol/L)	4.9	0.5 – 2.3
Procalcitonin (ng/mL)	0.1	0.03 – 0.49

**Figure 1:**
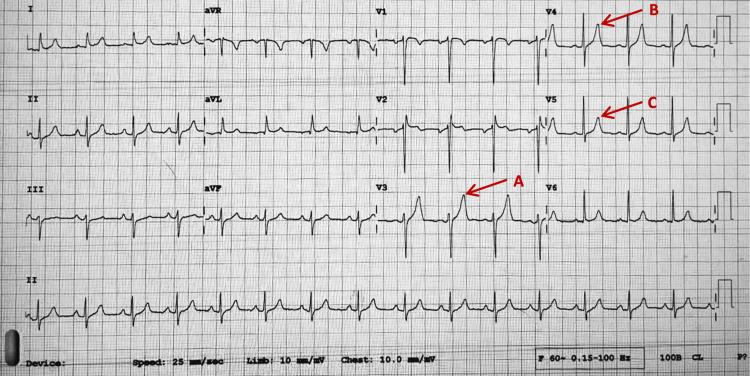
Electrocardiogram (A) Peak T wave in lead V3. (B) Peak T wave in lead V4. (C) Peak T wave in lead V5.

Electrocardiogram (EKG) showed normal sinus rhythm, ventricular rate = 92 bpm, PR interval = 149 milliseconds (ms), QRS duration = 84 ms, corrected QT interval (QTc) = 404 ms, and peaking of T waves noted in primarily the anterolateral leads between V3-V5 (Figure 1). Chest x-ray was unremarkable. Venous Doppler ultrasound of the right lower extremity showed no evidence of deep venous thrombosis.

The patient was started on aggressive intravenous (IV) hydration, starting with two liters of 0.9% normal saline upfront. Hyperkalemia was immediately treated with 25 grams (g) of 50% dextrose, 10 units of IV insulin, 1 g of IV calcium gluconate, 80 mg IV furosemide, 10 g oral (PO) sodium zirconium cyclosilicate, and 7.5 mg/0.5 mg nebulized albuterol/ipratropium x 2. Repeat chemistry showed a slight improvement of potassium (K = 7.0 meq/L) but worsening renal function (BUN = 38 mg/dL, Cr = 3.2 mg/dL). Given the limited resources of our hospital, the patient was immediately transferred to a tertiary hospital. There, he underwent an emergent four-compartment fasciotomy of his right lower extremity and required hemodialysis given subsequent worsening renal function presenting as acute renal failure (BUN = 115 mg/dL, Cr = 8.3 mg/dL). The patient’s surgical fasciotomy was successful and renal function returned to baseline after three sessions of hemodialysis. He was ultimately discharged home after a 10-day hospital stay with a wound vacuum device (wound VAC) to his right lower extremity with outpatient follow-up. The patient was seen again in our emergency department four months later ambulatory, neurovascularly intact, with a well-healing right lower extremity, for an unrelated complaint.

## Discussion

Our case report here describes a case of a heroin overdose that was complicated by acute lower extremity compartment syndrome, rhabdomyolysis, and acute renal failure. To date, very few case reports in the literature have demonstrated this phenomenon in a single patient. Our patient presented with a CK level of 212,845 U/L, which to our knowledge is the highest recorded value described in the literature for acute compartment syndrome in a single extremity [1-13].

As previously mentioned, the opioid epidemic in the United States underscores the importance of recognizing the various presentations that physicians may encounter in the emergency department. In acute opioid overdoses, patients often lack the appropriate cognitive function necessary to provide an appropriate, thorough history; thus necessitating a high level of clinical suspicion and complete physical examination in order to diagnose this rare phenomenon.

Several theories have been proposed to explain the pathophysiology of acute extremity compartment syndrome. The most widely accepted theory is the arteriovenous pressure gradient theory. Increased intra-compartmental pressure decreases arterial pressure while raising venous pressure thus decreasing the pressure gradient. This leads to decreased venous outflow and drainage of deoxygenated blood, ultimately compromising compartment perfusion [6].

In our case study, this pathophysiology is most analogous to that of a crush injury and prolonged immobilization. Prolonged immobilization in an unconscious state leads to rhabdomyolysis due to prolonged muscle compression, tissue damage, muscle ischemia, and leakage of intracellular contents through the capillary membrane into a closed space resulting in a rise in intra-compartmental pressures [7-10]. 

Rhabdomyolysis occurs when muscle damage is injured resulting in intracellular contents being released into the bloodstream. These contents include but are not limited to myoglobin, lactic acid, potassium, and calcium [11]. When myoglobin is released from skeletal muscle into the serum, it eventually becomes detectable in the urine (myoglobinuria). Myoglobin is directly toxic to the renal epithelial system as it can form renal cast depositions and is indirectly toxic to the renal system via renal vasoconstriction [4]. This can result in patients developing acute renal failure. Direct myotoxic effects of heroin and other illicit substances on muscle tissue have also been described. Indications for hemodialysis include refractory hyperkalemia, worsening renal function, and altered mental status [9].

In previous studies, CK levels have been found to be the most sensitive marker of myocyte injury. Normally, CK levels are between 45-260 U/L. Rhabdomyolysis can commonly result in CK levels ranging from 10,000 U/L to 200,000 U/L [4,12]. No other condition can cause such extreme levels of CK elevation. One review documented a mean CK level of 110,893 U/L in 29 cases of bilateral lower extremity compartment syndrome [1-13].

The electrolyte abnormalities can cause significant cardiac arrhythmias. While hypocalcemia, hypercalcemia, and hyperphosphatemia may result, hyperkalemia is the most dangerous. It can result in life-threatening arrhythmias, including ventricular fibrillation [2]. Patients with hyperkalemia often, but not always, demonstrate EKG changes in a progressive pattern once the potassium reaches > 5.5 meq/L. These progressive changes include peaked T waves (K = 5.5 - 6.5 meq/L), flattened P waves, shortened QT interval, prolonged PR interval (K = 6.5 - 7.0 meq/L), widening of the QRS complex, sine-wave pattern, ventricular fibrillation, and asystole (K = 7.0 - 9.0 meq/L) [6-9]. Patients with severe life-threatening hyperkalemia demonstrating EKG changes (peaked T waves) should immediately be given IV calcium gluconate to stabilize the cardiac membrane in addition to the other temporizing measures to shift potassium intracellularly as previously discussed [7-9].

Our patient was clinically diagnosed with acute lower extremity compartment syndrome given his clinical presentation and laboratory findings. While intra-compartmental pressures can be measured to aid in diagnosis, our facility lacked this equipment. Nonetheless, compartment syndrome remains a clinical diagnosis and emergent surgical consultation is warranted. Clinically, the patient presented with severe pain out of proportion, paresthesia, paralysis, and diminished pulses. His laboratory findings also supported that of severe rhabdomyolysis resulting in acute renal failure. 

While awaiting definitive treatment with emergency fasciotomy and hemodialysis at a tertiary transfer center, addressing the present comorbid conditions remained a priority for this patient. Aggressive IV fluid hydration was immediately initiated and the patient’s severe hyperkalemia was treated with temporizing measures as discussed previously. Prompt recognition and immediate intervention in the early stages of this patient’s condition likely contributed to his impressive recovery.

The patient was ultimately discharged home after a 10-day stay at the tertiary care facility and followed up appropriately with complete recovery of renal function and lower extremity motor/sensory function.

## Conclusions

This was an atypical, interesting case of a patient presenting initially as a heroin overdose whose course was complicated by several life-threatening conditions. Early recognition, treatment, and surgical consultation are the mainstay of treatment. Every patient presenting to the emergency department warrants a complete physical exam no matter the presenting complaint. Compartment syndrome remains a clinical diagnosis and emergent fasciotomy is the standard of care. Rhabdomyolysis is commonly caused by prolonged immobilization (crush injury) and requires early IV hydration to preserve renal function. In refractory and worsening cases of renal failure, emergent dialysis is required.
